# The National Cancer Institute’s Health Information National Trends Survey [HINTS]: a national cross-sectional analysis of talking to your doctor and other healthcare providers for health information

**DOI:** 10.1186/1471-2296-15-111

**Published:** 2014-06-06

**Authors:** Julie E Volkman, Tana M Luger, Kimberly LL Harvey, Timothy P Hogan, Stephanie L Shimada, Daniel Amante, D Keith McInnes, Hua Feng, Thomas K Houston

**Affiliations:** 1 eHealth Quality Enhancement Research Initiative, US Department of Veterans Affairs, 200 Springs Road, Bedford, MA 01730, USA; 2 University of Massachusetts Medical School, 368 Plantation Street, Worcester, MA 01605, USA; 3 Department of Health Policy and Management, Boston University School of Public Health, 715 Albany Street Talbot Building, T2W, Boston, MA 02118, USA

**Keywords:** Health information needs, Sources for health information, Doctor-patient communication, National cross-sectional survey

## Abstract

**Background:**

The need to understand preferred sources of health information remains important to providing patient-centered care. The Internet remains a popular resource for health information, but more traditional sources may still be valid for patients during a recent health need. This study sought to understand the characteristics of patients that turn to their doctor or healthcare provider first for a recent health or medical information need.

**Methods:**

Using the national cross-sectional survey, Health Information National Trend Study [HINTS], characteristics of those who sought a doctor or healthcare provider for a recent health information need were compared to other sources. Weighted survey responses from Cycle 1 and Cycle 2 of the HINTS survey were used for multivariable logistic regression.

**Results:**

A total 5,307 patient responses were analyzed. Overall, those who seek a doctor or healthcare provider first for a health need are female, 46–64 years, White non-Hispanic, educated, in good health and users of the Internet. Yet, adjusted logistic regressions showed that those who sought a doctor or healthcare provider first during a recent health information need compared to other sources were most likely to be 65+ years, in poor health, less educated and have health insurance.

**Conclusions:**

Patients who seek their doctor or healthcare provider first for health information rather than other sources of information represent a unique population. Doctors or healthcare providers remain an important resource for these patients during recent needs, despite the wide use of the Internet as a source of health information.

## Background

Health information access for patients is a key facet of the provision of patient-centered care [1]. Patients require appropriate health information in order to participate in treatment decisions and increase communication with their doctor or healthcare provider [1-5]. Doctors and healthcare providers recognize the importance of sources of health information, as improved patient access may increase patient satisfaction with care and lead to better health outcomes and self-management [4,6]. Doctors and healthcare providers have devoted time and resources to help understand patients’ health information needs and appropriate resources for them [6] to support patient chronic disease management [7] and to understand the value of different sources of health information for patients.

For several years, the Internet has become a frequent resource for health-related information for patients and their caregivers. In 2012, the Pew Internet & American Life Project reported 81% of U.S. adults use the Internet, and 72% said they have looked online for health information in the past 12 months [8]. Online health information is often sought by women more frequently than men, by more non-Hispanic Whites/Caucasians compared to Hispanics and African-Americans, more often by those with higher incomes and higher education levels, and by those who are employed [9-11]. Yet, the Internet does not remain the sole source of health information for patients. When asked to consider their last serious health issue and source of health information, either online or offline, 40% of U.S. adults surveyed in 2012 reported receiving information, care, or support from a doctor or other healthcare provider [8]. Additionally, patients traditionally trust their doctor or healthcare provider as a source of information, compared to other communication channels [12], and patients tend to solicit family, friends, or co-workers as reliable sources of information [13]. For example, racial minority groups tend to trust friends and family members for health information [14], and older adults prefer to rely on social sources of information as well [15]. Thus, in spite of its popularity, the Internet may not be the only resource for some patients when seeking health information. Understanding these preferences for patients is important for providing patient-centered care [1].

While the Internet remains a viable source of health information, it is clear that individuals have not completely eliminated more traditional approaches to information acquisition. We wished to gain insight into the health information habits of patients by understanding characteristics of patients turning towards a doctor or healthcare provider first when confronted with a recent health or medical information need. We analyzed responses to a national cross-sectional survey, the Health Information National Trend Survey (HINTS) sponsored by the National Cancer Institute, to understand where patients would turn to first during a recent medical need and the significant characteristics of individuals turning to a doctor or healthcare provider first compared to other information sources.

## Method

### Study design, sample and data collection

Data for our analyses were from the National Cancer Institute’s Health Information National Trends Survey (HINTS 2011–2013) (http://hints.cancer.gov), a national cross-sectional survey of the U.S. adult population (ages 18 years and older) assessing how individuals find, use and understand health information. Data were collected in two cycles: a) Cycle 1 from October 2011 to February 2012 (n = 3959) and Cycle 2 from October 2012 through January 2013 (n = 3630). Both cycles were administered through mailed questionnaires. The sample design for HINTS 2011–2013 was a two-stage, stratified sample. In the first stage, a stratified sample of addresses was selected from a file of residential addresses. In the second-stage, one adult was selected within each sampled household using one of two randomly assigned respondent selection conditions where either the adult with the next birthday or all adults within the household completed a questionnaire. The sampling frame consisted of a database of addresses used by Marketing Systems Group (MSG) to provide random samples of addresses [16]. The response rate for Cycle 1 was 36.7% and for Cycle 2 was 39.97% [16]. Extensive details on the study design, methods and sampling plan of HINTS have been published elsewhere [17,18].

### Participants

Combined data for HINTS 2011–2013 Cycle 1 and Cycle 2 resulted in a total of 7,589 individual respondents. Participants were included in the analyses if they were 18 years of age or older, had been asked about their most recent time seeking information about health or medical topics, and had provided any response to the question of interest (N = 5,307). We did not include in the analysis missing responses (n = 33), erroneous responses (n = 965) or responses for whom the question was not ascertained (n = 1,284).

### Measures

The HINTS survey includes 11 sections (A-K) across 125 questions. Sections include inquiries about looking for health information; using the Internet to find health information; health care; health, nutrition and physical activity; women and cancer; screening for cancer; beliefs about cancer; cancer history; looking for information about food and medical products; medical research and medical records; and household information.

Sources for a recent health information need was asked with the following question: “The most recent time you looked for information about health or medical topics, where did you go first?” Responses to this item (n = 5,307) were coded to create a dichotomous, dependent variable of “doctor or healthcare provider first” and “other sources” (books, brochures and pamphlets, cancer organization, family, friend/co-worker, Internet, library, magazines, newspapers, telephone information number, complementary, alternative or unconventional practitioner and all other specified sources). Questions were not asked related to the nature of the medical or health information need (see Additional file 1 for full HINTS survey).

Sociodemographic characteristics were assessed by inclusion of the following variables: age, gender, rural status, income, cancer history, general health, race/ethnicity, education, health insurance, Veteran status and Internet use and access to the Internet.

Age was categorized as 18–45; 46–64; 65–75; and 76–99 years according to the age distribution of respondents. Rural status was established using Census data as being completely rural or less than 2,500 urban population, adjacent or not adjacent to a metro area. Income level was derived from reported household pre-tax earnings from all sources within the past year and categorized as less than $20,000, $20,000 to less than $50,000 and equal to or greater than $50,000. Dichotomous cancer history was assessed with “Have you ever been diagnosed as having cancer?” General health status was measured with “In general, you would say your health is?” along the responses of Excellent, Very Good, Good, Fair and Poor. Race/ethnicity was categorized as non-Hispanic White, non-Hispanic black, Hispanic and all remaining responses collapsed as “other.” Health insurance was categorized as having indicated healthcare coverage (not including dental or vision) through an employer, purchased privately through an insurance company, Medicare/Medicaid or other government program, VA, Tricare, IHS or any other specified source. The derived variable of Veteran status was determined by those who responded as having ever served on active duty in the U.S. armed forces, military reserves or national guard and/or positive response to having ever received or enrolled for healthcare or medical services at the VA or having TRICARE or other military healthcare insurance coverage. Internet use was categorized as having a positive response to the item “Do you ever go on-line to access the Internet or World Wide Web, or to send/receive email?” Access to the Internet was assessed as dial-up telephone lines, broadband (DSL, cable or FIOS), cellular network (phone, 3G/4G) and/or wireless network (Wi-Fi). Access to other sources of health information was not asked in the survey (see Additional file 1).

### Analysis

Given the complex survey design of the HINTS study for both Cycles 1 and 2, we conducted a weighted analysis using procedures for analyzing sample survey data in SAS version 9.3 in order to calculate accurate population and subpopulation parameter estimates and confidence intervals for the U.S. adult population. We computed descriptive statistics including means and totals. Jackknife weights, provided by HINTS [17,18], were used for calculating standard errors. Multivariable logistic regression analysis was used to examine the association of first-sought health information sources with key sociodemographic characteristics. We used a cutoff of *p* < 0.05 to determine statistical significance for all analyses. The secondary analysis approved by the Department of Veterans Affairs Research and Development Committee.

## Results

Overall, health information seekers reported most frequently going first to the Internet (67.63%), followed by a doctor or healthcare provider first (15.66%), publications (books, brochures, magazines and newspapers) first (9.39%), and family/friends/co-workers first (4.95%) (See Table 1).

**Table 1:** First health information source (N = 5,307)

**Source**	**N**	**Weighted %**	**95% CI**
Internet	3,315	67.63	65.88 – 69.38
Doctor or healthcare provider	937	15.66	13.96 – 17.36
Publications (books, pamphlets, brochures, magazines and newspapers)	652	9.39	8.35 – 10.43
Family/friends/co-workers	235	4.95	4.03 – 5.87
Other	72	0.99	0.58 – 1.39
Telephone Service	40	0.61	0.34 – 0.89
Cancer Organization	17	0.26	0.08 – 0.44
Library	21	0.23	0.09 – 0.37
Complementary or alternative medicine practitioner	18	0.28	0.12 – 0.43

Missing = 857.

Those who sought a doctor or healthcare provider first for a recent health information need were female (53.81%), 46–64 years (34.66%), were non-Hispanic White (58.93%), earned $50,000 and above annually (34.14%), had some college education (31.83%) and more than college education (20.39%), had health insurance (85.86%) and were not a Veteran (80.64%). The majority did not live in rural areas (97.68%), did not have a cancer history (86.12%), and reported having very good (31.08%) to good (34.63%) general health. More than half (61.81%) currently used the Internet and accessed it through Broadband (64.29%), although it was not their “first sought” health source for a recent medical need (See Table 2).

**Table 2:** Characteristics health information seekers choosing doctor or healthcare provider first (n = 937)

	**N**	**Weighted %**	**95% CI**
**Gender**			
Male	385	43.16	38.61 – 47.71
Female	531	53.81	49.38 – 58.23
Missing	21	3.03	0.47 – 5.58
**Age**			
18–45	150	28.62	23.12-34.13
46–64	347	34.66	30.02-39.31
65–75	241	18.14	15.31-20.97
76–99	179	14.71	12.17-17.24
Missing	20	3.87	0–8.13
**Rurality**			
Yes (8–9)	21	2.32	1.11-3.53
No (1–7)	916	97.68	96.47-98.89
Missing	~	~	~
**Cancer history**			
Yes	186	13.50	11.25-15.74
No	746	86.12	83.83-88.40
Missing	5	.39	0–0.78
**General health**			
Excellent	71	8.71	5.77-11.64
Very good	302	31.08	26.47-35.68
Good	347	34.63	30.00-39.25
Fair	141	16.47	12.70-20.24
Poor	52	6.48	2.81-10.15
Missing	24	2.64	1.36-3.92
**Income**			
Less than and $20,000	234	26.40	21.01 – 31.79
$20,000 to $49,999	258	27.10	22.20 – 32.00
$50 k or above	311	34.14	30.68 – 38.22
Missing	134	12.35	8.96 – 15.75
**Race/Ethnicity**			
White, NH	532	58.93	54.15 – 63.72
African-American, NH	143	9.17	6.94 – 11.41
Hispanic	111	16.36	11.63 – 21.08
Other, NH	63	6.74	3.89 – 9.58
Missing	88	8.80	6.23 – 11.37
**Education**			
Less than high School	118	23.11	17.22 – 28.99
High school graduate	240	23.01	18.84 – 27.17
Some college	292	31.83	26.98 – 36.69
College or above	266	20.39	17.08 – 23.69
Missing	21	1.67	0.82 – 2.51
**Health insurance**			
Yes	853	85.86	81.56 – 90.15
No	69	13.02	8.83 – 17.21
Missing	15	1.12	0.41 – 1.84
**Veteran status**			
Veteran	139	11.45	8.87 – 14.02
Non-Veteran	735	80.64	77.11 – 84.17
Missing	63	7.91	4.84 – 10.99
**Internet use**			
Yes	550	61.81	57.11 – 66.50
No	386	38.17	33.48 – 42.85
Missing	1	0.03	0.00 – 0.08
**Access to internet**			
Telephone line	52	5.09	2.67-7.53
Broadband (DSL, cable, or FIOS)	359	37.47	32.33-42.61
Cellular network (phone, 3G/4G)	166	22.29	16.53-28.04
Wireless network (Wi-Fi)	274	31.30	26.00-36.60
Missing	12	5.12	0.00-10.84

Differences did emerge in the characteristics of those who sought a doctor or healthcare provider first for a recent health information need compared to individuals that cited other source options (e.g., Internet, books, friends/family/co-workers, etc.) as their first choice. Results from the adjusted logistic regression showed that those who sought a doctor or healthcare provider first were most likely to be 65–75 years [OR = 3.05 (95% CI, 2.13-4.35), p = .0001) and 75–99 years [OR = 2.88 (95% CI, 1.89-4.38), p = .006], less likely to have good health [OR = .88 (95% CI = .53-1.47), p = .03] and more likely to be in poor health [OR = 3.99 (95% CI, .80-20.02), p = .05], less likely to be a college graduate or more [OR = 0.35 (95% CI, 0.17-0.75), p = .0009], more likely to have health insurance [OR = 1.72 (95% CI,1.03-2.88), p = .04], and less likely to use the Internet [OR = 0.40 (95% CI, 0.29-0.56), p < .0001] compared to those who sought other sources of information first during a recent health need (e.g., Internet, books, friends/family/co-workers, etc.). Gender and rural status did not significantly differ between doctor or healthcare provider first seekers and seekers of other sources (See Table 3). Unadjusted differences for cancer history, income, race, and veteran status were mitigated in the adjusted model. Stratifying the sample by Internet use did not yield different results regarding significant characteristics or significant interactions among the variables of interest.

**Table 3:** Characteristics of health information seekers choosing doctor or healthcare provider first (n = 937) compared to other sources (Total N = 4,370)

	**Doctor or healthcare provider first N (weighted %)**	**Other sources N (weighted %)**	**Unadjusted OR (95% CI), p-value**	**Adjusted OR (95% CI), p-value**
**Gender**				
Male	385 (14.94)	1,592 (85.06)	1.00 --	1.00 --
Female	531 (15.93)	2,695 (84.07)	0.93 (0.75 – 1.15), p = .47	1.00 (0.71 – 1.39), p = .98
**Age**				
18–45	150 (9.41)	1,541 (90.59)	1.00 --	1.00--
46–64	347 (15.52)	1,880 (84.48)	**1.77 (1.30-2.41), p < .0001**	1.74 (1.28-2.36), p = .18
65–75	241 (29.23)	601 (70.77)	**3.98 (3.01-5.25), p < .0001**	**3.05 (2.13-4.35), p = .0001**
76-99	179 (40.76)	262 (59.24)	**6.63 (4.72-9.31), p < .0001**	**2.88 (1.89-4.38), p = .006**
**Rurality**				
No	916 (15.55)	4,301 (84.45)	1.00 --	1.00--
Yes	21 (22.38)	69 (77.62)	1.57 (0.86-2.86), p = .15	1.20 (0.46-3.13), p = .71
**Cancer history**				
No	746 (14.72)	3,816 (85.28)	1.00 --	1.00--
Yes	186 (25.98)	535 (74.02)	**2.03 (1.63-2.53), p < .0001**	1.21 (0.91-1.62), p = .19
**General health**				
Excellent	71 (11.59)	558 (88.41)	1.00 --	1.00--
Very good	302 (12.85)	1,630 (87.15)	**1.13 (0.72-1.75), p < .0001**	0.95 (0.59-1.54), p = .08
Good	347 (15.78)	1,526 (84.22)	**1.43 (0.94-2.18), p = .02**	**0.88 (0.53-1.47), p = .03**
Fair	141 (21.69)	497 (78.31)	2.11 (1.33-3.36), p = .26	0.98 (0.55-1.73), p = .24
Poor	52 (43.23)	90 (56.77)	**5.81 (2.31-14.64), p = .0002**	**3.99 (0.80-20.02), p = .05**
**Income**				
Less than $20,000	234 (22.57)	692 (77.43)	1.00 --	1.00 --
$20,000 to $49,999	258 (16.33)	1,149 (83.67)	0.67 (0.47 – 0.96), p = .95	0.89 (0.59 – 1.33), p = .73
$50,000 and above	311 (11.41)	2,116 (88.59)	**0.44 (0.33 – 0.60), p < .0001**	0.87 (0.55 – 1.36), p = .63
**Race**				
Non-Hispanic White	532 (13.93)	2,812 (86.07)	1.00 --	1.00 --
Non-Hispanic Black	143 (15.04)	581 (84.96)	1.09 (0.81 – 1.48), p = .53	1.11 (0.75 – 1.64), p = .69
Hispanic	111 (21.27)	461 (78.73)	**1.67 (1.12 – 2.48), p = .04**	1.25 (0.79 – 1.97), p = .81
Other	63 (15.35)	271 (84.65)	1.12 (0.65 – 1.93), p = .75	1.45 (0.70-3.02), p = .48
**Education**				
Less than High School	118 (37.56)	218 (62.44)	1.00 --	1.00 --
High School Graduate	240 (19.92)	689 (80.08)	0.41 (0.25 – 0.68), p = .29	0.63 (0.33-1.23), p = .51
Some College	292 (13.99)	1,318 (86.01)	**0.27 (0.17 – 0.44), p = .002**	0.52 (0.26-1.05), p = .35
College Grad. or More	266 (9.11)	2,071 (90.89)	**0.17 (0.11 – 0.26), p < .0001**	**0.35 (0.17-0.75), p = .0009**
**Health insurance**				
No	69 (12.01)	563 (87.99)	1.00 --	1.00 --
Yes	853 (16.34)	3,767 (83.66)	1.43 (0.96 – 2.13), p = .07	**1.72 (1.03-2.88), p = .04**
**Veteran status**				
Non-Veteran	735 (14.43)	3,780 (85.57)	1.00 --	1.00 --
Veteran	139 (19.48)	459 (80.52)	**1.44 (1.06 – 1.94), p = .02**	1.04 (0.72-1.49), p = .84
**Internet use**				
No	386 (42.35)	547 (57.65)	1.00 --	1.00 --
Yes	550 (11.27)	3,823 (88.73)	**0.17 (0.14 – 0.22), p < .0001**	**0.40 (0.29-0.56), p < .0001**

Note: Significant relationships are bolded.

OR = Odds ratio; 95% CI = Confidence Interval.

## Discussion

### Overview of main findings

Understanding sources of health information is important given the prevalence of health information available online, and the implications of using online health information on health behaviors and health outcomes related to patient activation, health misinformation, and more. Results from our study suggest seeking information from a doctor or healthcare provider is a “not-so-close second” behind the Internet for health or medical information needs. Compared to other sources of health information, doctors and other healthcare providers still represent an important, trusted group for health or medical information, in particular for older patients, those with less education, patients who have health insurance and those who perceive themselves as being in poor health. Interestingly, those who turn to their doctor first were still Internet users in general, but chose to solicit a doctor or healthcare provider for health information instead of the Internet for a recent health or medical need. Doctors and healthcare providers thus still represent a very viable source of information for patients, specifically for a unique population.

### Comparison with existing literature

The HINTS analyses contrast with other studies which have found gender differences in the use of the Internet for health information as well as income-based, racial, and geographic differences [19] in access to the Internet [8,10,11,20]. In particular, Pew Internet and American Life Survey [8], using a smaller sample size of 3,014 adults living in the U. S., found that women, adults over 50 years old, non-Hispanic whites, and those with some college were more likely to get information from a physician when experiencing “a serious health issue or significant change in your health.” Interestingly, these characteristics are comparable to the initial characteristics of our population from our findings. Yet, the Pew questions concern a more specific, serious health issue compared to the HINTS questionnaire which assesses a recent health or medical need. It is possible that gender and racial differences are found when health information is needed for a substantial reason, but age and education are more powerful predictors of a preference to rely on a physician first for general health information. For example, older adults and those who are less educated tend to prefer to be less involved overall in medical decisions [21], which could lend itself to relying on “expert” physicians for general health information.

Furthermore, patients who reported going to their doctor or health care provider first for a recent health information need were most frequently over 65 years old. Older adults are more likely to have complex medical profiles including multiple co-morbidities [22], making it necessary to synthesize health information about a variety of conditions. Consequently, a doctor or healthcare provider may be a first resource for health information needs as they are familiar with the complexities of multiple conditions. In addition, less educational attainment has been shown to be associated with more self-reported health information needs [23], making relying on the physician’s expertise perhaps more appealing in order to acquire the desired information. Health literacy [24] may also be a driving factor in the relationship between education and health information needs. Health literacy concerns a patient’s ability to acquire and understand health information [24] and is associated with lower educational attainment [25,26]. Thus, those with less education may have difficulty acquiring and processing information from other sources, preferring instead to rely on their physician. However, it is unclear how long HINTS respondents may have been seeing their doctor or healthcare provider for specific conditions or their health literacy level, which may have influenced the results for this sub-sample of individuals. Finally, those with health insurance were more likely to solicit information from a physician, suggesting that information from a doctor or healthcare provider may be a convenient and viable option.

Considering the differences by age, education and insurance, our results offer unique implications for those that did *not* select a doctor or healthcare provider as their first choice as a source during a recent health need. While our results are cross-sectional and causation cannot be inferred, it does offer suggestions about how online sources and other social sources (friends, family, co-workers, etc.) may affect the traditional information relationships between patients and their doctors and healthcare providers. For those younger, more educated, uninsured individuals that selected the Internet or other social sources, there may be concerns about the quality and accuracy of the information they find [27,28], in addition to how they assimilate the information. Potentially, this information, if misinterpreted through communication outside of the patient-healthcare provider relationship or due to the complexities of multiple conditions, may put patients at risk.

### Strengths and limitations

HINTS draws its strength from a reliance on multiple methods in order to reduce non-response [29], which was typical of a postal survey. The selected sample was sent an initial mailing of the questionnaire, a reminder card, and up to three additional questionnaire mailings. This also improved response rates from previous telephone administered waves of the HINTS survey. In addition, our analyses use the HINTS-recommended weights in order to adjust for non-response and provide more accurate parameter estimates. Nevertheless, our findings are not without limitations. The HINTS survey did not include information about the types of health information sought, which prevents us from probing if patients rely more on their physician for specific types of information regarding their conditions or other health needs. Additionally, we do not have knowledge of the type of doctor or healthcare provider, or length of the relationship, which could be significant influences regarding choice. Second, while we attempted to utilize rurality and type of Internet access as indicators of access to information, these may not reflect the true availability of health information to responders or their ability to process said information. Finally, health literacy was not assessed in the HINTS survey which could provide further explanation for group differences. Despite these limitations, the results do offer suggestions for future research and implications for sources for health information.

### Future research

Participants in the HINTS survey may represent a unique sample of information-seekers as our lack of findings for income or racial differences might indicate. Further exploration of a preference for physician-provided health information in additional national samples for both serious and general health information needs may identify other significant factors. In addition, health literacy is likely an important construct which affects a preference for doctor-provided information. Future work will want to examine patient preferences for information across health literacy abilities in order to disentangle patients who avoid conducting their own health information searches rather than those who prefer to rely on the expertise of their physician regardless of their literacy level. With the recent initiation of the Patient Protection and Affordable Care Act in 2010 [30], additional research may want to see how changes in insurance regulations may change patient patterns in preferred sources of information. Finally, providing appropriate, effective health information depends on the particular patient’s needs. Recent movements in developing patient-reported outcome measures such as the NIH’s PROMIS [31] can provide physicians with standardized patient information which may help physicians to tailor health information provision for patients. Investigations into how patient-reported outcomes might inform the type and volume of information that patients require from physicians will be important.

## Conclusions

The changing healthcare landscape makes it important to understand the needs of patients regarding their health information needs and sources of information. This study found that older, less educated patients with health insurance were more likely to rely on doctors or healthcare providers as a reliable source for health and medical topics. Older, less educated patients may require more information provision during face-to-face visits, as they may not desire to seek additional information via online or other sources. Continuing education training in patient-centered communication [32], which involves health information provision based upon the patient’s needs and values and increasing patient-provider trust, may also support doctors and healthcare providers in giving effective health information to these patients in order to achieve optimal outcomes.

## Competing interests

The authors declare that they have no competing interests.

## Authors’ contributions

JV and TKH conceived of the study, participated in the coordination of analyses and drafted the manuscript. TL participated in the draft of the manuscript and an interpretation of the results. KLLH analyzed the results and participated in the draft of the manuscript. TPH, SLS, DKM, DA and HF made substantial contributions to the conception and design of the analyses, acquisition of the data and interpretation of the data. All authors read and approved the final manuscript.

## Pre-publication history

The pre-publication history for this paper can be accessed here:

http://www.biomedcentral.com/1471-2296/15/111/prepub

## Supplementary Material

Additional file 1 Health Information National Trends Survey. Click here for file
