# Supplementary material for: The National Cancer Institute’s Health Information National Trends Survey [HINTS]: a national cross-sectional analysis of talking to your doctor and other healthcare providers for health information
Source: BMC Fam Pract. 2014 Jun 6;15:111. doi: 10.1186/1471-2296-15-111 (PMC4055386; doi:10.1186/1471-2296-15-111)
Supplement: Additional file 1 — Health Information National Trends Survey. [file 1471-2296-15-111-S1.pdf]

National Institutes of Health
U.S. Department of Health and Human Services

OMB # 0925-0538
Expiration Date: October 31, 2014

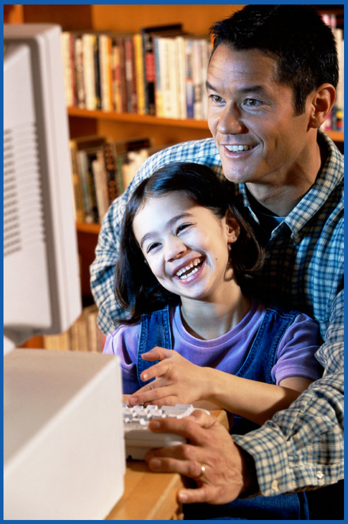

Health Information
National Trends Survey

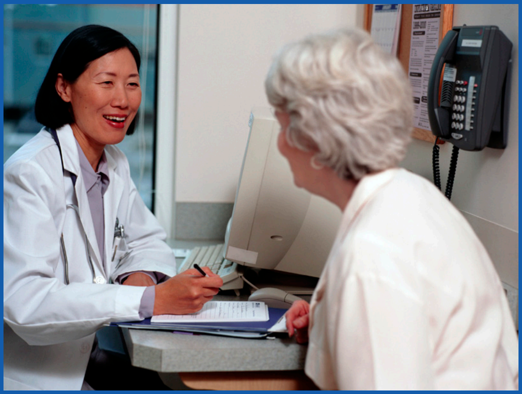

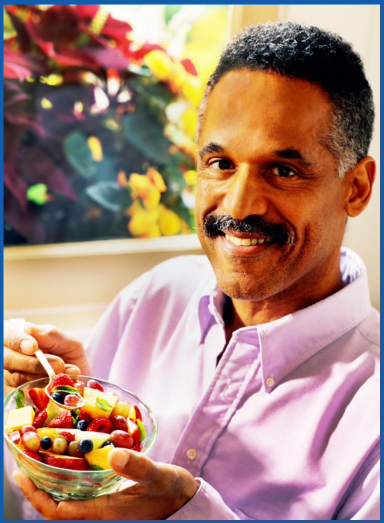

START HERE:

1. Is there more than one person age 18 or older living in this household?

AdultsInHH

1 Yes

0 No → **GO TO A1 on the next page**

2. Including yourself, how many people age 18 or older live in this household?

MailHHAdults

3. **The adult with the next birthday should complete this questionnaire.** This way, across all households, HINTS will include responses from adults of all ages.

4. Please write the first name, nickname or initials of the adult with the next birthday. This is the person who should complete the questionnaire.

HHAdultNextBirthday

Si prefiere recibir la encuesta en español, por favor llame 1-888-738-6812

STATEMENT OF PRIVACY: Collection of this information is authorized by The Public Health Service Act, Sections 411 (42 USC 285 a) and 412 (42 USC 285a-1.a and 285a1.3). Rights of study participants are protected by The Privacy Act of 1974. Participation is voluntary, and there are no penalties for not participating or withdrawing from the study at any time. Refusal to participate will not affect your benefits in any way. The information collected in this study will be kept private under the Privacy Act and will only be seen by people authorized to work on this project. The report summarizing the findings will not contain any names or identifying information. Identifying information will be destroyed when the project ends.

NOTIFICATION TO RESPONDENT OF ESTIMATED BURDEN: Public reporting burden for this collection of information is estimated to average 30 minutes per response, including the time for reviewing instructions, searching existing data sources, gathering and maintaining the data needed, and completing and reviewing the collection of information. **An agency may not conduct or sponsor, and a person is not required to respond to, a collection of information unless it displays a currently valid OMB control number.** Send comments regarding this burden estimate or any other aspect of this collection of information, including suggestions for reducing this burden to: NIH, Project Clearance Branch, 6705 Rockledge Drive, MSC 7974, Bethesda, MD 20892-7974, ATTN: PRA (0925-0538). Do not return the completed form to this address.

UniqueID

A: Looking For Health Information

A1. Have you ever looked for information about health or medical topics from any source?
SeekHealthInfo
- 1 Yes
- 0 No → **GO TO A6 in the next column**

A2. The most recent time you looked for information about health or medical topics, where did you go first?

Mark ☒ only one.
WhereSeekHeathInfo
- 1 Books InfoBook
- 2 Brochures, pamphlets, etc. InfoBroch
- 3 Cancer organization InfoCorg
- 4 Family InfoFam
- 5 Friend/Co-Worker InfoFriend
- 6 Doctor or health care provider InfoDoc
- 7 Internet InfoWww
- 8 Library InfoLib
- 9 Magazines InfoMag
- 10 Newspapers InfoPpr
- 11 Telephone information number InfoTino
- 12 Complementary, alternative, or unconventional practitioner InfoCaup
- 91 Other-Specify → WhereSeekHealthInfo_OS
  InfoOther
  InfoMulti

A3. Did you look or go anywhere else that time?
LookElsewhere
- 1 Yes
- 0 No

A4. The most recent time you looked for information about health or medical topics, who was it for?
WhoLookingFor
- 1 Myself
- 2 Someone else
- 3 Both myself and someone else

A5. Based on the results of your most recent search for information about health or medical topics, how much do you agree or disagree with each of the following statements?

	Strongly agree	Somewhat agree	Somewhat disagree	Strongly disagree
LotOfEffort<br>a. It took a lot of effort to get the information you needed..................................	1	2	3	4
Frustrated<br>b. You felt frustrated during your search for the information............................	1	2	3	4
ConcernedQuality<br>c. You were concerned about the quality of the information............................	1	2	3	4
TooHardUnderstand<br>d. The information you found was hard to understand............................	1	2	3	4

A6. Overall, how confident are you that you could get advice or information about health or medical topics if you needed it?
ConfidentGetHealthInf
- 1 Completely confident
- 2 Very confident
- 3 Somewhat confident
- 4 A little confident
- 5 Not confident at all

A7. In general, how much would you trust information about health or medical topics from each of the following?

	Not at all	A little	Some	A lot
TrustDoctor<br>a. A doctor..................................	1	2	3	4
TrustFamily<br>b. Family or friends....................	1	2	3	4
TrustNewsmag<br>c. Newspapers or magazines......	1	2	3	4
TrustRadio<br>d. Radio....................................	1	2	3	4
TrustInternet<br>e. Internet..................................	1	2	3	4
TrustTelevision<br>f. Television...............................	1	2	3	4
TrustGov<br>g. Government health agencies...	1	2	3	4
TrustCharities<br>h. Charitable organizations..........	1	2	3	4
TrustReligiousOrgs<br>i. Religious organizations and leaders..............................	1	2	3	4

UniqueID

41419

A8. Imagine that you had a strong need to get information about health or medical topics. Where would you go first?

Mark ☒ only one.
StrongNeedHealthInfo

- 1 Books FinfoBook
- 2 Brochures, pamphlets, etc. FinfoBroch
- 3 Cancer organization FinfoCorg
- 4 Family FinfoFam
- 5 Friend/Co-Worker FinfoFriend
- 6 Doctor or health care provider FinfoDoc
- 7 Internet FinfoWww
- 8 Library FinfoLib
- 9 Magazines FinfoMag
- 10 Newspapers FinfoPpr
- 11 Telephone information number FinfoTino
- 12 Complementary, alternative, or unconventional practitioner FinfoCaup
- 91 Other-Specify → StrongNeedHealthInfo_OS

FinfoOther
FinfoMulti

A9. Have you ever looked for information about cancer from any source?
SeekCancerInfo

- 1 Yes
- 0 No

A10. How much attention do you pay to information about health or medical topics from each of the following sources?

	None	A little	Some	A lot
Attention_OnlineNews<br>a. In online newspapers.................	1	2	3	4
Attention_PrintNews<br>b. In print newspapers..................	1	2	3	4
Attention_HealthNews<br>c. In special health or medical magazines or newsletters..........	1	2	3	4
Attention_Internet<br>d. On the Internet.........................	1	2	3	4
Attention_Radio<br>e. On the radio.............................	1	2	3	4
Attention_LocalTV<br>f. On local television news programs.................................	1	2	3	4
Attention_NatTV<br>g. On national or cable television news programs..........	1	2	3	4

A11. Genetic tests that analyze your DNA, diet and lifestyle for potential health risks are currently being marketed by companies directly to consumers. Have you heard or read about these genetic tests?
HeardGeneticTest

- 1 Yes
- 0 No

B: Using the Internet to Find Information

B1. Do you ever go on-line to access the Internet or World Wide Web, or to send and receive e-mail?
UseInternet

- 1 Yes
- 0 No → **GO TO C1 on the next page**

B2. When you use the Internet, do you access it through...

	Yes	No
Internet_DialUp<br>a. A regular dial-up telephone line...............	1	0
Internet_BroadBnd<br>b. Broadband such as DSL, cable or FiOS...	1	0
Internet_Cell<br>c. A cellular network (e.g., phone, 3G/4G)...	1	0
Internet_WiFi<br>d. A wireless network (Wi-Fi).......................	1	0

B3. Do you access the Internet any other way?
Internet_Other

- 1 Yes - Specify → Internet_OtherOS
- 0 No

B4. In the past 12 months, have you used the Internet to look for health or medical information for yourself?
InternetHealthInfoSelf

- 1 Yes
- 0 No

B5. Is there a specific Internet site you like to go to for health or medical information?
InternetHealthInfoSite

- 1 Yes
- 0 No → **GO TO B7 on the next page**

B6. Specify which Internet site you especially like as a source of health or medical information:

InternetHealthInfoSite_OS

41419

B7. In the last 12 months, have you used the Internet for any of the following reasons?

	Yes	No
IntRsn_InfQuitSmoking<br>a. Looked for information about quitting smoking	1	0
IntRsn_BuyMedicine<br>b. Bought medicine or vitamins on-line	1	0
IntRsn_SupportGroup<br>c. Participated in an on-line support group for people with a similar health or medical issue	1	0
IntRsn_TalkDoctor<br>d. Used e-mail or the Internet to communicate with a doctor or doctor's office	1	0
IntRsn_DietWebsite<br>e. Used a website to help you with your diet, weight, or physical activity	1	0
IntRsn_HCProviderSearch<br>f. Looked for a health care provider	1	0
IntRsn_PDADownload<br>g. Downloaded health-related information to a mobile device, such as an MP3 player, cell phone, tablet computer or electronic book device	1	0
IntRsn_SocNetworkSite<br>h. Visited a "social networking" site, such as "Facebook" or "LinkedIn" to read and share about medical topics	1	0
IntRsn_Blog<br>i. Wrote in an on-line diary or "blog" (i.e., Web log) about any type of health topic	1	0
IntRsn_TrackedPHR<br>j. Kept track of personal health information such as care received, test results, or upcoming medical appointments	1	0
IntRsn_HealthInfSE<br>k. Looked for health or medical information for someone else	1	0

B8. Have you done anything else health-related on the Internet?

OthInternetHealthRelated

1 Yes-Specify → OthInternetHealthRelated_OS

0 No

C: Your Health Care

C1. Not including psychiatrists and other mental health professionals, is there a particular doctor, nurse, or other health professional that you see most often?

RegularProvider

1 Yes

0 No

C2. Do you have any of the following health insurance or health coverage plans:

	Yes	No
HCCoverage_Insurance<br>a. Insurance through a current or former employer or union (of you or another family member)	1	0
HCCoverage_Private<br>b. Insurance purchased directly from an insurance company (by you or another family member)	1	0
HCCoverage_Medicare<br>c. Medicare	1	0
HCCoverage_Medicaid<br>d. Medicaid, Medical Assistance, or any kind of government-assistance plan for those with low incomes or disability	1	0
HCCoverage_Tricare<br>e. TRICARE or other military health care	1	0
HCCoverage_VA<br>f. VA (including those who have ever used or enrolled for VA health care)	1	0
HCCoverage_IHS<br>g. Indian Health Service	1	0

C3. Do you have any other health care coverage plan for yourself (please do not include dental or vision plans)?

HCCoverage_Other

1 Yes-Specify → HCCoverage_OtherOS

0 No

C4. About how long has it been since you last visited a doctor for a routine checkup? A routine checkup is a general physical exam, not an exam for a specific injury, illness, or condition.

MostRecentCheckup

1 Within past year (anytime less than 12 months ago)

2 Within past 2 years (1 year but less than 2 years ago)

3 Within past 5 years (2 years but less than 5 years ago)

4 5 or more years ago

-8 Don't know

5 Never

C5. In the past 12 months, not counting times you went to an emergency room, how many times did you go to a doctor, nurse, or other health professional to get care for yourself?

FreqGoProvider

0 None → **GO TO D1 on the next page**

1 1 time

2 2 times

3 3 times

4 4 times

5 5-9 times

6 10 or more times

C6. The following questions are about your communication with all doctors, nurses, or other health professionals you saw during the past 12 months...

How often did they do each of the following:

	Always	Usually	Sometimes	Never
ChanceAskQuestions a. Give you the chance to ask all the health-related questions you had?....................................	1	2	3	4
FeelingsAddressed b. Give the attention you needed to your feelings and emotions?......	1	2	3	4
InvolvedDecisions c. Involve you in decisions about your health care as much as you wanted?.................................	1	2	3	4
UnderstoodNextSteps d. Make sure you understood the things you needed to do to take care of your health?.......................	1	2	3	4
ExplainedClearly e. Explain things in a way you could understand?.........................	1	2	3	4
SpentEnoughTime f. Spend enough time with you?.......	1	2	3	4
HelpUncertainty g. Help you deal with feelings of uncertainty about your health or health care?.............................	1	2	3	4

C7. In the past 12 months, how often did you feel you could rely on your doctors, nurses, or other health care professionals to take care of your health care needs?
DrTakeCareNeeds
1 Always
2 Usually
3 Sometimes
4 Never

C8. Overall, how would you rate the quality of health care you received in the past 12 months?
QualityCare
1 Excellent
2 Very good
3 Good
4 Fair
5 Poor

C9. In the past 12 months, have you talked to a doctor, nurse, or other health professional about any kind of health information you have gotten from the Internet?
TalkedDoctor
1 Yes
0 No → **GO TO D1 in the next column**

C10. In the past 12 months when you talked with a health care professional, how interested were they in hearing about the information you found on-line?
DoctorInterested
1 Very interested
2 Somewhat interested
3 A little interested
4 Not at all interested

D: Your Health, Nutrition and Physical Activity

D1. In general, would you say your health is...
GeneralHealth
1 Excellent,
2 Very good,
3 Good,
4 Fair, or
5 Poor?

D2. Over the past 2 weeks, how often have you been bothered by any of the following problems?

	Nearly every day	More than half the days	Several days	Not at all
LittleInterest a. Little interest or pleasure in doing things..........................	1	2	3	4
Hopeless b. Feeling down, depressed or hopeless............................	1	2	3	4
Nervous c. Feeling nervous, anxious or on edge...............................	1	2	3	4
Worrying d. Not being able to stop or control worrying....................	1	2	3	4

D3. Overall, how confident are you about your ability to take good care of your health?
OwnAbilityTakeCareHealth
1 Completely confident
2 Very confident
3 Somewhat confident
4 A little confident
5 Not confident at all

41419

D4. How often in the past 12 months would you say you were worried or stressed about having enough money to buy nutritious meals?
StressedMoneyNutritiousMeals
- 1 Always
- 2 Usually
- 3 Sometimes
- 4 Rarely
- 5 Never

D5. When available, how often do you use menu information on calories in deciding what to order?
UseMenuCalorieInfo
- 1 Always
- 2 Often
- 3 Sometimes
- 4 Rarely
- 5 Never

D6. About how many cups of fruit (including 100% pure fruit juice) do you eat or drink each day?
Fruit
- 0 None
- 1 ½ cup or less
- 2 ½ cup to 1 cup
- 3 1 to 2 cups
- 4 2 to 3 cups
- 5 3 to 4 cups
- 6 4 or more cups

1 cup of fruit could be:
- 1 small apple
- 1 large banana
- 1 large orange
- 8 large strawberries
- 1 medium pear
- 2 large plums
- 32 seedless grapes
- 1 cup (8 oz.) fruit juice
- ½ cup dried fruit
- 1 inch-thick wedge of watermelon

D7. About how many cups of vegetables (including 100% pure vegetable juice) do you eat or drink each day?
Vegetables
- 0 None
- 1 ½ cup or less
- 2 ½ cup to 1 cup
- 3 1 to 2 cups
- 4 2 to 3 cups
- 5 3 to 4 cups
- 6 4 or more cups

1 cup of vegetables could be:
- 3 broccoli spears
- 1 cup cooked leafy greens
- 2 cups lettuce or raw greens
- 12 baby carrots
- 1 medium potato
- 1 large sweet potato
- 1 large ear of corn
- 1 large raw tomato
- 2 large celery sticks
- 1 cup of cooked beans

D8. How much sugar-sweetened soda or pop do you usually drink each day? Do not include diet sodas or diet pop.
RegularSoda
- 0 None
- 1 12 ounces (1 can) or less
- 2 13 to 24 ounces (2 cans)
- 3 25 to 36 ounces (3 cans)
- 4 37 to 48 ounces (4 cans)
- 5 more than 48 ounces

D9. <u>In a typical week</u>, how many days do you do any physical activity or exercise of at least moderate intensity, such as brisk walking, bicycling at a regular pace, and swimming at a regular pace?
TimesModerateExercise
- 0 None → **GO TO D11 below**
- 1 1 day per week
- 2 2 days per week
- 3 3 days per week
- 4 4 days per week
- 5 5 days per week
- 6 6 days per week
- 7 7 days per week

D10. On the days that you do any physical activity or exercise of at least moderate intensity, how long are you typically doing these activities?

Write a number in one box below.
HowLongModerateExerciseMn

☐☐ Minutes ☐☐ Hours

HowLongModerateExerciseHr

D11. In a typical week, outside of your job or work around the house, how many days do you do leisure-time physical activities specifically designed to strengthen your muscles such as lifting weights or circuit training (do not include cardio exercise such as walking, biking, or swimming)?
TimesStrengthTraining
- 0 None
- 1 1 day per week
- 2 2 days per week
- 3 3 days per week
- 4 4 days per week
- 5 5 days per week
- 6 6 days per week
- 7 7 days per week

41419

D12. Over the past 30 days, in your leisure time, how many hours per day, on average, did you sit and watch TV or movies, surf the web, or play computer games? Do not include "active gaming" such as Wii.
AverageDailyTVGames
[ ][ ] Hours per day

D13. About how tall are you without shoes?
Height_Feet Height_Inches
[ ] Feet ***and*** [ ][ ] Inches

D14. About how much do you weigh, in pounds, without shoes?
Weight
[ ][ ][ ] Pounds

D15. How many times in the past 12 months have you used a tanning bed or booth?
TanningBed
- 0 0 times
- 1 1 to 2 times
- 2 3 to 10 times
- 3 11 to 24 times
- 4 25 or more times

D16. When you are outside for more than one hour on a warm, sunny day, how often do you wear sunscreen?
Sunscreen
- 1 Always
- 2 Often
- 3 Sometimes
- 4 Rarely
- 5 Never
- 99 Do not go out on sunny days

D17. Have you smoked at least 100 cigarettes in your entire life?
Smoke100
- 1 Yes
- 0 No → **GO TO D19 in the next column**

D18. How often do you now smoke cigarettes?
SmokeNow
- 1 Everyday
- 2 Some days
- 3 Not at all

D19. A drink of alcohol is 1 can or bottle of beer, 1 glass of wine, 1 can or bottle of wine cooler, 1 cocktail, or 1 shot of liquor.

During the past 30 days, how many days per week did you have at least one drink of any alcoholic beverage?
DrinksPerWeek
- 0 0 days → **GO TO D21 below**
- 1 1 day
- 2 2 days
- 3 3 days
- 4 4 days
- 5 5 days
- 6 6 days
- 7 7 days

D20. During the past 30 days, on the days when you drank, about how many drinks did you drink on the average?
DrinksPerDay
[ ][ ] Drink(s)

D21. How much sleep do you usually get...

	Hours	Minutes
a. SleepWorkdayHr,SleepWorkdayMn On a workday or school day (i.e., weekday)?........................	[ ][ ]	[ ][ ]
b. SleepWeekendHr,SleepWeekendMn On a non-work or non-school day (i.e., weekend)?.................	[ ][ ]	[ ][ ]

E: Women and Cancer

E1. Are you male or female?
GenderC
- 1 Male → **GO TO F1 on the next page**
- 2 Female

E2. Has a doctor ever told you that you could choose whether or not to have the Pap test?
DrTalkPapTest
- 1 Yes
- 0 No

41419

E3. How long ago did you have your most recent Pap test to check for cervical cancer?
WhenPapTest
- 1 A year ago or less
- 2 More than 1, up to 2 years ago
- 3 More than 2, up to 3 years ago
- 4 More than 3, up to 5 years ago
- 5 More than 5 years ago
- 6 I have never had a Pap test

E4. A mammogram is an x-ray of each breast to look for cancer.

Has a doctor ever told you that you could choose whether or not to have a mammogram?
DrTalkMammo
- 1 Yes
- 0 No

E5. When did you have your most recent mammogram to check for breast cancer, if ever?
WhenMammogram
- 1 A year ago or less
- 2 More than 1, up to 2 years ago
- 3 More than 2, up to 3 years ago
- 4 More than 3, up to 5 years ago
- 5 More than 5 years ago
- 6 I have never had a mammogram

F: Screening for Cancer

F1. A vaccine to prevent HPV infection is available and is called the HPV shot, cervical cancer vaccine, GARDASIL®, or Cervarix®.

Has a doctor or other health care professional ever talked with you about the HPV shot or vaccine?
EverTalkedHPVShot
- 1 Yes
- 0 No

F2. There are a few different tests to check for colon cancer. These tests include:

A **colonoscopy** – For this test, a tube is inserted into your rectum and you are given medication that may make you feel sleepy. After the procedure, you need someone to drive you home.

A **sigmoidoscopy** – For this test, you are awake when the tube is inserted into your rectum. After the test you can drive yourself home.

A **stool blood test** – For this test, you collect a stool sample at home, and then provide it to a doctor or lab for testing.

Has a doctor ever told you that you could choose whether or not to have a test for colon cancer?
DrTalkColCaTest
- 1 Yes
- 0 No

F3. Have you ever had a test to check for colon cancer?
EverHadColCaTest
- 1 Yes
- 0 No

F4. (Females **GO TO G1** on the next page. Males continue with **F4**.) The following questions are about discussions doctors or other health care professionals may have with their patients about the PSA test that is used to look for prostate cancer.

Has a doctor ever told you that you could choose whether or not to have the PSA test?
DrTalkPSATest
- 1 Yes
- 0 No

F5. Have you ever had a PSA test?
EverHadPSATest
- 1 Yes
- 0 No
- 3 Not sure

F6. Has a doctor or other health care professional ever told you that some doctors recommend the PSA test and others do not?
SomeRecommendPSATest
- 1 Yes
- 0 No
- 3 Not sure

F7. Has a doctor or other health care professional ever told you that no one is sure if using the PSA test actually saves lives?
NotSurePSATest
- 1 Yes
- 0 No
- 3 Not sure

G: Beliefs About Cancer

▶ Think about cancer in general when answering the questions in this section.

G1. How likely are you to get cancer in your lifetime?
ChanceGetCancer
- 1 Very unlikely
- 2 Unlikely
- 3 Neither unlikely nor likely
- 4 Likely
- 5 Very likely

G2. Compared to other people your age, how likely are you to get cancer in your lifetime?
CompareChanceGetCancer
- 1 Very unlikely
- 2 Unlikely
- 3 Neither unlikely nor likely
- 4 Likely
- 5 Very likely

G3. How worried are you about getting cancer?
FreqWorryCancer
- 1 Not at all
- 2 Slightly
- 3 Somewhat
- 4 Moderately
- 5 Extremely

G4. How much do you agree or disagree with each of the following statements?

	Strongly agree	Somewhat agree	Somewhat disagree	Strongly disagree
EverythingCauseCancer<br>a. It seems like everything causes cancer..........................	1	2	3	4
PreventNotPossible<br>b. There's not much you can do to lower your chances of getting cancer..........................	1	2	3	4
TooManyRecommendations<br>c. There are so many different recommendations about preventing cancer, it's hard to know which ones to follow..........	1	2	3	4

H: Your Cancer History

H1. Have you ever been diagnosed as having cancer?
EverHadCancer
- 1 Yes
- 0 No → **GO TO H4 on the next page**

H2. What type of cancer did you have?

Mark ☒ all that apply.

- 1 CaBladder Bladder cancer
- 1 CaBone Bone cancer
- 1 CaBreast Breast cancer
- 1 CaCervical Cervical cancer (cancer of the cervix)
- 1 CaColon Colon cancer
- 1 CaEndometrial Endometrial cancer (cancer of the uterus)
- 1 CaHeadNeck Head and neck cancer
- 1 CaHodgkins Hodgkin's lymphoma
- 1 CaLeukemia Leukemia/Blood cancer
- 1 CaLiver Liver cancer
- 1 CaLung Lung cancer
- 1 CaMelanoma Melanoma
- 1 CaNonHodgkin Non-Hodgkin lymphoma
- 1 CaOral Oral cancer
- 1 CaOvarian Ovarian cancer
- 1 CaPancreatic Pancreatic cancer
- 1 CaPharyngeal Pharyngeal (throat) cancer
- 1 CaProstate Prostate cancer
- 1 CaRectal Rectal cancer
- 1 CaRenal Renal (kidney) cancer
- 1 CaSkin Skin cancer, non-melanoma
- 1 CaStomach Stomach cancer
- 1 CaOther Other-Specify → CaOther_OS

Cancer_Cat

41419

H3. At what age were you first told that you had cancer?
WhenDiagnosedCancer

[ ][ ][ ] Age

H4. Have any of your family members ever had cancer?
FamilyEverHadCancer

1 Yes
0 No
4 Not sure

I: Looking for Information about Food and Medical Products

I1. Do you agree or disagree with the following statements:

	Agree	Disagree	No opinion
FoodInfoClear<br>a. Information about foods is easy to understand	1	2	3
FoodInfoHelpful<br>b. Information about foods helps me make the right food choices	1	2	3
FoodInfoAvailable<br>c. I can easily find information about the foods I eat	1	2	3

▶ The next few questions are about over-the-counter drugs.

I2. Do you buy any over-the-counter drugs (you don't need a doctor's prescription) for yourself or someone else?
BuyOTCDrugs

1 Yes
0 No → **GO TO I7 in the next column**

I3. Do you agree or disagree with the following statements:

	Agree	Disagree	No opinion
OTCDrugBenefitsClear<br>a. Information about the benefits of over-the-counter drugs is easy to understand	1	2	3
OTCDrugBenefitsHelpful<br>b. Information about the benefits of over-the-counter drugs helps me decide whether to buy a drug	1	2	3
OTCDrugBenefitsAvailable<br>c. I can easily find information about the benefits of the over-the-counter drugs I may buy	1	2	3

I4. Do you agree or disagree with the following statements:

	Agree	Disagree	No opinion
OTCDrugRisksClear<br>a. Information about the risks of over-the-counter drugs is easy to understand	1	2	3
OTCDrugRisksHelpful<br>b. Information about the risks of over the-counter drugs helps me decide whether to buy a drug	1	2	3
OTCDrugRisksAvailable<br>c. I can easily find information about the risks of the over-the-counter drugs I may buy	1	2	3

I5. When you first buy over-the-counter drugs, how often do you read the directions and warnings label?
OTCDrugReadInfo

1 Always
2 Often
3 Sometimes
4 Rarely
5 Never

I6. "The directions and warnings label on over-the-counter drugs is easy to understand." Do you...
OTCDrugInfoEasy

1 Agree,
2 Disagree, or
3 Have no opinion?

I7. Do you agree or disagree with the following statements:

	Agree	Disagree	No opinion
OTCAdsBenefits<br>a. Ads for over-the-counter drugs tell me enough about the benefits of using the drugs	1	2	3
OTCAdsNegatives<br>b. Ads for over-the-counter drugs tell me enough about their negative side-effects	1	2	3

I8. Do you agree or disagree with the following statements:

	Agree	Disagree	No opinion
OTCSaferThanRx<br>a. Over-the-counter drugs are safer than prescription drugs	1	2	3
OTCLessEffectiveThanRx<br>b. Over-the-counter drugs are less effective than prescription drugs	1	2	3

The next few questions are about prescription drugs.

I9. Do you buy any prescription drugs for yourself or anyone else?
BuyRxDrugs
1 Yes
0 No → **GO TO I14 in the next column**

I10. When you first buy drugs that a doctor prescribes, how often do you read the directions and warnings that come with the drug?
RxDrugReadInfo
1 Always
2 Often
3 Sometimes
4 Rarely
5 Never

I11. What would you do if a prescription drug you purchased for yourself or someone else was recalled? Would you:

		**Yes**	**No**
a.	RxDrugRecall_StopTaking<br>Stop taking it at once................................	1	0
b.	RxDrugRecall_KeepUsing<br>Keep using the drug................................	1	0
c.	RxDrugRecall_Ignore<br>Pay no attention to the recall....................	1	0
d.	RxDrugRecall_OnGuard<br>Be on your guard....................................	1	0
e.	RxDrugRecall_VisitWebsite<br>Go on the manufacturer's website.............	1	0
f.	RxDrugRecall_ContactManuf<br>Contact the manufacturer........................	1	0
g.	RxDrugRecall_ContactDoctor<br>Contact the doctor/nurse/other medical professional................................	1	0
h.	RxDrugRecall_ContactPharm<br>Talk to the pharmacist..............................	1	0
i.	RxDrugRecall_Unsure<br>Unsure..	1	0

I12. Would you do anything else if a prescription drug you purchased for yourself or someone else was recalled?
RxBrugRecall_Other
1 Yes-Specify → RxDrugRecall_OtherOS
0 No

I13. "The directions and warnings that come with prescription drugs are easy to understand." Do you...
RxDrugInfoEasy
1 Agree,
2 Disagree, or
3 Have no opinion?

I14. Do you agree or disagree with the following statements:

		Agree	Disagree	No opinion
a.	RxDrugAdsBenefits<br>Ads for prescription drugs tell me enough about the benefits of using the drugs..	1	2	3
b.	RxDrugAdNegatives<br>Ads for prescription drugs tell me enough about their negative side-effects....................................	1	2	3

The next few questions are about medical products.

I15. At any time in the last 12 months, have you purchased any common household medical product for yourself or for someone else in your household, such as bandages (e.g., Band-Aids®), a thermometer, an electronic toothbrush or a pregnancy test kit?
BuyHHMP
1 Yes
0 No

I16. At any time in the last 12 months have you purchased a medical product for yourself or for someone else in your household to help care for a chronic condition, such as a walker, blood glucose kit, hearing aid, blood pressure cuff, contact lenses or prescription eye glasses?
BuyMP
1 Yes
0 No

I17. At any time in the last 12 months have you purchased any other type of medical product for yourself or for someone else in your household, such as a powered wheelchair, motorized scooter, or hospital bed?
BuyOtherMP
1 Yes
0 No

! If you answered "yes" to I15 or I16 or I17 then continue to I18 on the next page.

If you answered "no" to all 3 questions then GO TO Question I20 on the next page.

I18. When you first buy a medical product, how frequently do you read the directions and warnings that come with it?

By medical product, we mean the kinds of medical products you included when answering I15, I16 and I17.

MPReadInfo

- 1 Always
- 2 Often
- 3 Sometimes
- 4 Rarely
- 5 Never

I19. "Directions and warnings that come with medical products are easy to understand." Do you...

MPInfoEasy

- 1 Agree,
- 2 Disagree, or
- 3 Have no opinion?

I20. Do you agree or disagree with the following statements:

		Agree	Disagree	No opinion
	MPAdsBenefits			
a.	Ads for common medical products tell me enough about the benefits of using these products......................	1	2	3
	MPAdsNegatives			
b.	Ads for common medical products tell me enough about the risks of using these products..........................	1	2	3

I21. You may have heard about some recent recalls on medical products. Examples of products that have recently been recalled are stents, pacemakers, infant apnea monitors, and automated external defibrillators (AEDs).

What would you do if any medical product that you or someone you love depended on was recalled? Would you....

		Yes	No
	MPRecall_StopUsing		
a.	Have it removed/stop using it	1	0
	MPRecall_ContactDoctor		
b.	Contact the doctor	1	0
	MPRecall_ContactManuf		
c.	Go on the manufacturer's website/ Contact the manufacturer	1	0
	MPRecall_Replace		
d.	Have it replaced/Find a substitute	1	0
	MPRecall_KeepUsing		
e.	Keep using it/Keep it	1	0
	MPRecall_NoChange		
f.	Make no change	1	0
	MPRecall_Unsure		
g.	Unsure/Don't know	1	0

I22. Would you do anything else if a medical product that you or someone you love depended on was recalled?

MPRecall_Other

- 1 Yes-Specify → MPRecall_OtherOS
- 0 No

I23. Did you ever visit the Food and Drug Administration's website (www.FDA.gov)?

VisitFDAWebsite

- 1 Yes → **GO TO I26 below**
- 0 No

I24. Why haven't you visited the FDA website?

Mark ☒ all that apply.

- FDASite_NoCompInternet 1 I don't own a computer (no Internet access)
- FDASite_NoReasonToVisit 1 I don't have a reason to visit the site
- FDASite_PreferOtherSites 1 I prefer other sites
- FDASite_DidntKnowAboutSite 1 I didn't know about the FDA site
- FDASite_DontTrustGovtSites 1 I don't trust government websites
- FDASite_DontTrustFDA 1 I don't trust the FDA
- FDASite_HardToNavigate 1 It's too hard to find information on the FDA website

I25. Is there any other reason you have not visited the FDA website?

FDASite_Other

- 1 Yes-Specify → FDASite_OtherOS
- 0 No

GO TO J1 on the next page

I26. On your most recent visit, did you find the information you were looking for?

FDASite_FoundInfo

- 1 Yes
- 0 No

I27. How easy or hard was it to find the information you were looking for?

FDASite_EaseOfUse

- 1 Very easy
- 2 Easy
- 3 Neither easy nor hard
- 4 Hard
- 5 Very hard

J: Medical Research and Medical Records

J1. As far as you know, do any of your doctors or other health care providers maintain your medical information in a computerized system?
ProviderMaintainEMR
1 Yes
0 No

J2. Please indicate how important each of the following statements is to you.

ShareEMR
AccessPHR

	Very important	Somewhat important	Not at all important
a. Doctors and other health care providers should be able to share your medical information with each other electronically..................	1	2	3
b. You should be able to get to your own medical information electronically..................................	1	2	3

J3. How confident are you that safeguards (including the use of technology) are in place to protect your medical records from being seen by people who aren't permitted to see them?

Having safeguards (including the use of technology) in place has to do with the security of your medical records.
ConfidentInfoSafe
1 Very confident
2 Somewhat confident
3 Not confident

J4. How confident are you that you have some say in who is allowed to collect, use and share your medical information?

Having a say in who can collect, use and share your medical information has to do with the privacy of your records.
ConfidentControlInfo
1 Very confident
2 Somewhat confident
3 Not confident

J5. Have you ever kept information from your health care provider because you were concerned about the privacy or security of your medical record?
WithheldInfoPrivacy
1 Yes
0 No

J6. If your medical information is sent by fax from one health care provider to another, how concerned are you that an unauthorized person would see it?
FaxedInfoSafe
1 Very concerned
2 Somewhat concerned
3 Not concerned

J7. If your medical information is sent electronically from one health care provider to another, how concerned are you that an unauthorized person would see it? (Electronically means from computer to computer, instead of by telephone, mail, or fax machine).
ElectInfoSafe
1 Very concerned
2 Somewhat concerned
3 Not concerned

K: You and Your Household

K1. What is your age?
Age
[ ][ ][ ] Years old

K2. What is your current occupational status?

Mark ☒ only one.
OccupationStatus
1 Employed Employed
2 Unemployed Unemployed
3 Homemaker Homemaker
4 Student Student
5 Retired Retired
6 Disabled Disabled
91 Other-Specify → OccupationStatus_OS
OtherOcc
MultiOcc

41419

K3. Have you ever served on active duty in the U.S. Armed Forces, military Reserves or National Guard? Active duty does not include training in the Reserves or National Guard, but DOES include activation, for example, for the Persian Gulf War.

ActiveDutyArmedForces

- [1] Yes, now on active duty
- [2] Yes, on active duty in the last 12 months but not now
- [3] Yes, on active duty in the past, but not in the last 12 months
- [4] No, training for Reserves or National Guard only — **GO TO K4**
- [5] No, never served in the military — **GO TO K4**

K3a. In the past 12 months, have you received some or all of your health care from a VA hospital or clinic?

ReceivedCareVA

- [1] Yes, all my health care
- [2] Yes, some of my health care
- [3] No, no VA health care received

K4. What is your marital status?

MaritalStatus

- [1] Married
- [2] Living as married
- [3] Divorced
- [4] Widowed
- [5] Separated
- [6] Single, never been married

K5. What is the highest grade or level of schooling you completed?

Education

- [1] Less than 8 years
- [2] 8 through 11 years
- [3] 12 years or completed high school
- [4] Post high school training other than college (vocational or technical)
- [5] Some college
- [6] College graduate
- [7] Postgraduate

K6. Were you born in the United States?

BornInUSA

- [1] Yes → **GO TO K8 in the next column**
- [0] No

K7. In what year did you come to live in the United States?

YearCameToUSA

[ ][ ][ ][ ] Year

K8. How comfortable do you feel speaking English?

ComfortableEnglish

- [1] Completely comfortable
- [2] Very comfortable
- [3] Somewhat comfortable
- [4] A little comfortable
- [5] Not at all comfortable

K9. Are you Hispanic or Latino?

Hispanic

- [1] Yes
- [0] No

K10. Which one or more of the following would you say is your race?

Mark ☒ one or more boxes.

- AmerInd [1] American Indian/Alaska Native
- Asian [1] Asian
- Black [1] Black/African American
- PacIsl [1] Native Hawaiian/Other Pacific Islander
- White [1] White

Race_Cat

K11. Including yourself, how many people live in your household?

TotalHousehold

[ ][ ] Number of people

K12. Including yourself, please mark the gender and write in the age and month of birth for each adult 18 years of age or older living at this address.

	Gender	Age	Month Born (01-12)
HHAdultGender1 Adult 1	[1] Male [2] Female	HHAdultAge1	HHAdultMOB1
HHAdultGender2 Adult 2	[1] Male [2] Female	HHAdultAge2	HHAdultMOB2
HHAdultGender3 Adult 3	[1] Male [2] Female	HHAdultAge3	HHAdultMOB3
HHAdultGender4 Adult 4	[1] Male [2] Female	HHAdultAge4	HHAdultMOB4
HHAdultGender5 Adult 5	[1] Male [2] Female	HHAdultAge5	HHAdultMOB5

41419

K13. How many children under the age of 18 live in your household?

ChildrenInHH

[ ][ ] Number of children under 18

K14. Do you currently rent or own your home?

RentOrOwn

1 Own
2 Rent
3 Occupied without paying monetary rent

K15. Does anyone in your family have a working cell phone?

CellPhone

1 Yes
0 No

K16. Is there at least one telephone inside your home that is currently working and is not a cell phone?

PhoneInHome

1 Yes
0 No

K17. Thinking about members of your family living in this household, what is your combined annual income, meaning the total pre-tax income from all sources earned in the past year?

IncomeRanges

1 $0 to $9,999
2 $10,000 to $14,999
3 $15,000 to $19,999
4 $20,000 to $34,999
5 $35,000 to $49,999
6 $50,000 to $74,999
7 $75,000 to $99,999
8 $100,000 to $199,999
9 $200,000 or more

K18. Did you complete this survey all in one sitting, or did you do it in more than one sitting?

MailStartStopSurvey

1 I completed the survey all in one sitting.
2 I completed the survey in more than one sitting.

K19. Did anyone help you complete this survey?

MailSomeoneInRoom

1 Yes
0 No

K20. About how long did it take you to complete the survey?

Write a number in one box below.

MailSurveyTime_Min MailSurveyTime_Hrs

[ ][ ] Minutes [ ][ ] Hours

K21. At which of the following types of addresses does your household currently receive residential mail?

Mark ☒ all that apply.

TypeOfAddressA
1 A street address with a house or building number
TypeOfAddressB
1 An address with a rural route number
TypeOfAddressC
1 A U.S. post office box (P.O. Box)
TypeOfAddressD
1 A commercial mail box establishment (such as Mailboxes R Us, and Mailboxes Etc.)

Thank you!

▶ Please return this questionnaire in the postage-paid envelope at your earliest convenience.

▶ If you have lost the envelope, mail the completed questionnaire to:

HINTS Study, TC 1046F

Westat

1600 Research Boulevard

Rockville, MD 20850
